# The impact of meteorological variables on *Salmonella* bacteraemia in Mysuru District, Karnataka State, India: a retrospective time-series analysis

**DOI:** 10.1177/20499361251389056

**Published:** 2025-10-27

**Authors:** Naveen Manchal, Mahadevaiah N. Sumana, Megan K. Young, Maria Eugenia Castellanos, Peter Leggat, Oyelola A. Adegboye

**Affiliations:** Public Health and Tropical Medicine, College of Public Health, Medical and Veterinary Sciences, James Cook University, Townsville, QLD, Australia; Department of Microbiology, JSS Medical College and Hospital, JSS Academy of Higher Education and Research, Mysuru, Karnataka, India; Metro North Public Health Unit, Metro North Hospital and Health Service, Brisbane, QLD, Australia; School of Medicine and Dentistry, Griffith University, QLD, Australia; School of Public Health, University of Queensland, St Lucia, QLD, Australia; Public Health and Tropical Medicine, College of Public Health, Medical and Veterinary Sciences, James Cook University, Townsville, QLD, Australia; Australian Institute of Tropical Health and Medicine, James Cook University, Townsville, QLD, Australia; World Health Organization Collaborating Centre for Vector-Borne and Neglected Tropical Diseases, James Cook University, Townsville, QLD, Australia; Public Health and Tropical Medicine, College of Public Health, Medical and Veterinary Sciences, James Cook University, Townsville, QLD, Australia; Australian Institute of Tropical Health and Medicine, James Cook University, Townsville, QLD, Australia; World Health Organization Collaborating Centre for Vector-Borne and Neglected Tropical Diseases, James Cook University, Townsville, QLD, Australia; Public Health and Tropical Medicine, College of Public Health, Medical and Veterinary Sciences, James Cook University, Townsville, QLD 4811, Australia; Australian Institute of Tropical Health and Medicine, James Cook University, Townsville, QLD 4811, Australia; World Health Organization Collaborating Centre for Vector-Borne and Neglected Tropical Diseases, James Cook University, Townsville, QLD 4811, Australia; Menzies School of Health Research, Charles Darwin University, Darwin, NT 0810, Australia

**Keywords:** bloodstream infections, *Salmonella* bacteraemia, temperature

## Abstract

**Background:**

*Salmonella* species are major pathogens responsible for gastroenteritis and typhoid fever. In 2017, *Salmonella enterocolitis* caused over 95 million cases of diarrhoea and 50,000 deaths globally, with India bearing more than 50% of the typhoid burden.

**Objectives:**

To test the association of monthly mean and maximum temperature, precipitation and absolute humidity with the incidence of *Salmonella* bacteraemia in a metropolitan city in South India.

**Design:**

A retrospective time-series analysis.

**Methods:**

This study employed a retrospective time-series analysis to evaluate the influence of meteorological variables, including temperature, absolute humidity and precipitation, on *Salmonella* bacteraemia in a metropolitan city in South India.

**Results:**

Between 2014 and 2019, a total of 492 blood culture-confirmed cases of *Salmonella* bacteraemia were identified in Mysuru, India. *S. typhi* was predominantly among younger patients, while non-typhoidal *Salmonella* was more frequent in older age groups. Resistance was highest to nalidixic acid (84%), with a rising trend in ciprofloxacin resistance. Increased mean temperature (lags 1–3 months) and absolute humidity were positively associated with *Salmonella* bacteraemia, while temperature variability was protective, and monsoon rainfall significantly increased the risk. Cumulative exposure–response analyses further showed elevated risks at higher humidity (>26 g/m³), temperatures (>34°C) and extreme precipitation (>250 mm), although confidence intervals were wide and most associations did not reach statistical significance.

**Conclusion:**

This single-centre retrospective time-series analysis has demonstrated that meteorological variables impact the incidence of *Salmonella* bacteraemia, which could lead to increased antibiotic use and contribute to the development of resistance.

## Plain language summary

Impact of meteorological variables on Salmonella

Using a retrospective time-series approach, our study evaluates how temperature, absolute humidity, and precipitation influence Salmonella infections in a metropolitan setting in South India, where typhoid and non-typhoidal salmonellosis remain significant public health challenges. While associations between meteorological variables and Salmonella bacteraemia cases were observed, our findings underscore emerging antibiotic resistance trends, particularly high resistance (84%) to Nalidixic acid and a concerning increase in Ciprofloxacin resistance.

## Introduction

*Salmonella* is a major pathogen that contributes significantly to the global burden of foodborne gastroenteritis and enteric fever. The Global Burden of Diseases, Injuries, and Risk Factors study estimated that *Salmonella enterocolitis* contributed to 95.1 million cases of diarrhoea, 50,771 deaths, and 3.1 million Disability-Adjusted Life Years (DALYs) in 2017.^1–3^ Over 2500 serotypes of *Salmonella* have been identified, and *S. enterica* sub sp *enterica* is the commonest human pathogen.^4^ Typhoidal serovars (typhi, and paratyphi A, B and C) cause enteric fever, while non-typhoidal *Salmonella* (NTS) causes gastroenteritis. All species of *Salmonella* can cause bacteraemia, with an NTS incidence of approximately 5%.^4^ Humans are the only reservoirs for typhoidal serotypes, with the transmission occurring primarily through the faecal-oral route. By contrast, animals are the main reservoir for NTS. The emergence of multi-drug-resistant (MDR) *Salmonella* worldwide is a growing concern.^5^

India bears more than 50% of the global *Typhoid* burden, predominantly in younger age groups.^6^ The Surveillance for Enteric Fever in India (SEFI) estimated that the incidence in children up to 14 years was between 12 and 1622 per 100,000 child-years.^6^ Ninety-eight percent of these isolates were not susceptible to ciprofloxacin, highlighting escalating antimicrobial resistance.^6^ A study conducted in Bangladesh estimated that of all *Salmonella* bacteraemia, the prevalence of MDR *Salmonella* was 85%,^7^ Additionally, there is an emerging trend of NTS infections in India.^8^

The burden of *Salmonella* infections, particularly enteric fever, may worsen with climate change. Studies have shown that rising ambient temperatures and precipitation increase the risk of enteric infections.^9–13^ In their modelling study, Chua et al.^14^ found that if current climate trends continue, mortality attributable to heat will mainly be caused by *Salmonella typhi* and *Shigella s*p. This trend will pose unique challenges to a country like India, which is experiencing a rising incidence of heat waves and floods.^15^ The impact would be greatest on the lowest socio-economic groups, who have limited access to health care and limited means of adapting to the changing climate.^16^ Furthermore, invasive NTS infections are increasing, with 92% diagnosed on blood culture and higher rates in the elderly population.^17^

While the link between climate variables and *Salmonella* gastroenteritis is well known, there is limited evidence on bacteraemia. Understanding this relationship is crucial for predicting future disease burden and planning public health strategies. This study aimed to examine the relationship between ambient temperature, rainfall and humidity and the occurrence of *Salmonella* bacteraemia in a metropolitan city in South India. The results can be used to predict the incidence of severe infection with this major enteric pathogen in the coming years if the current trends in climate change continue.

## Methods

### Study population and study site

**Figure 1.**
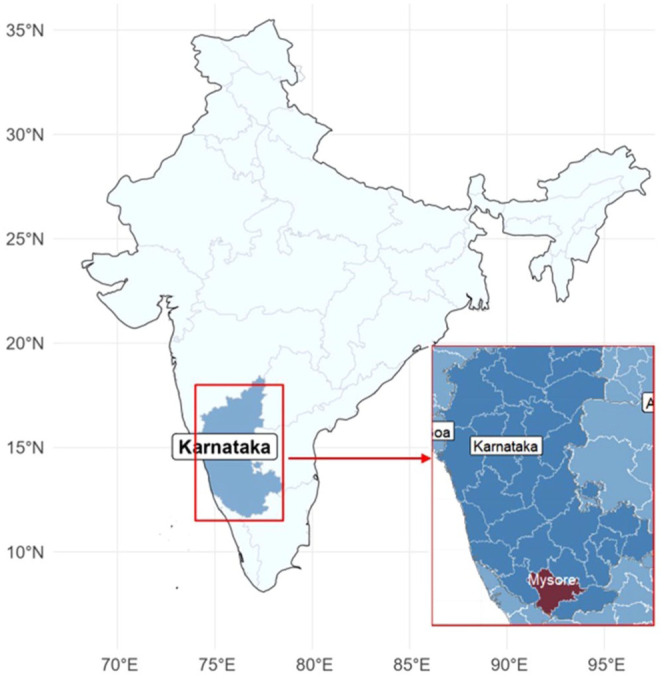
Map of India showing the study area, Mysuru district.

This retrospective time-series analysis was conducted in a tertiary centre in Mysuru, India. Mysuru is a metropolitan city in the southern Indian state of Karnataka with a population of 1.2 million as of 2020 (Figure 1). The city has a hot, semi-arid climate, characterised by summers from March to May, monsoons from June to October and winters from November to February. The city’s primary water sources are the Cauvery River and groundwater. Recently, the city has experienced significant climate change with rising ambient temperatures.^18^ The study site was a major private teaching hospital and university with advanced research facilities and laboratories. A large section of the population, regardless of socio-economic background, accesses the hospital.

Time-series data on all positive *Salmonella* blood culture isolates from January 2014 to December 2019 were extracted from microbiology laboratory software at Jagadguru Sri Shivarathreeshwara (JSS) University. Each isolate was assigned a unique identification number to ensure patient data remained anonymous. Isolates were then organised by pathogen. The outcome measure for this study was the aggregated monthly number of *Salmonella* bacteraemia cases. Other variables collected include de-identified patient demographic data, such as age and sex. The reporting of this study conforms to the Strengthening the Reporting of Observational Studies in Epidemiology (STROBE) statement.^19^

### Exposure variables

The exposure variables for this study were monthly mean and maximum temperatures (°C), monthly mean precipitation (mm), monthly relative humidity (%) and the number of days with flood waves greater than the 95th percentile of river discharge. These variables were obtained from the Indian Bureau of Meteorology for the Mysuru weather station.

### Statistical analysis

We first described the study population and the characteristics of the patients with *Salmonella* blood isolates. Temporal trends in meteorological variables were visualised with the monthly incidence of *Salmonella*. To explore potential associations between these variables, we conducted a cross-correlation analysis. Univariate quasi-Poisson regression was also fitted to examine the relationship between meteorological factors and *Salmonella* cases at various lags, based on cross-correlation analysis.

Given the non-linear nature of the outcome and the delayed effects on *Salmonella* bacteraemia, we employed distributed lag non-linear models (DLNM) to examine the strength of these associations.^9,12,20–24^ The association between these variables, infection incidence and lagged days was modelled via a quasi-Poisson regression framework with cross-basis parameterisation to estimate the exposure–lag–response surface. This approach allowed us to calculate the exposure–response function.

We also introduced a bi-dimensional cross-basis function between meteorological variables (temperature, humidity and precipitation) and *Salmonella* bacteraemia (exposure–lag–response) to capture the non-linear and delayed relationship. For absolute humidity, the exposure–response function was modelled using a natural cubic B-spline with knots placed at the 10th, 75th and 90th percentiles to capture the non-linear exposure–response relationship. By contrast, the lag response was modelled using two interior knots at equally spaced values in the log scale over a 2-month period to account for potential delayed effects. The temperature exposure–response function was modelled using a B-spline with equally spaced knots, and the lag–response used a natural cubic spline with logarithmic knots, with a maximum lag of 3 months. This allowed for flexible, non-linear and delayed temperature effects on the outcome. The exposure–response function for precipitation was modelled using a linear function with a 1-month lag period. All models account for seasons and temporal trends and were implemented in R version 4.0.3.

## Results

### *Salmonella* cases

**Figure 2.**
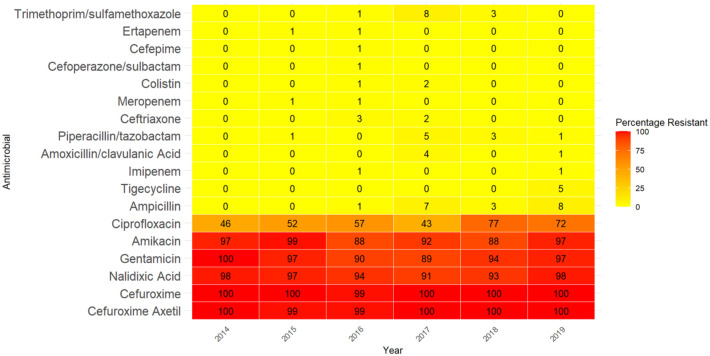
Trends in antimicrobial susceptibility profiles among 492 blood *Salmonella* isolates in Mysuru, India, 2014–2019. The values inside each cell represent the percentage of resistant isolates for a given antimicrobial in that year.

Between 2014 and 2019, 492 positive blood culture isolates of *Salmonella* species were identified. *S. typhi* was most common in the younger age groups, while NTS was most common in the older age groups (Table 1). The cohort consisted of 67% males. *Salmonella* blood isolates showed resistance of >50% to a third (6/18) of antimicrobials: gentamicin, amikacin, cefuroxime axetil, cefuroxime, nalidixic acid and ciprofloxacin (Figure 2).

**Table 1.** Profile and characteristics of patients with *Salmonella* bacteraemia during the study period.

Characteristic	Overall, *N* = 492	*S. typhi* *N* = 391	*S. paratyphi* A, *N* = 85	*S. typhimurium* , *N* = 7	Other NTS, *N* = 7	*S. paratyphi B* *N* = 2
Age group, *n* (%)						
0–20	279 (56.8%)	232 (83.1%)	43 (15.4%)	2 (0.7%)	1 (0.3%)	1 (0.3%)
21–40	135 (27.4%)	101 (74.8%)	32 (23.7%)	2 (1.4%)	1 (0.3%)	(0%)
41–60	55 (11.2%)	41 (74.5%)	10 (18.1%)	0 (0%)	3 (5.4%)	1 (1.8%)
60+	22 (0.04%)	16 (72.7%)	2 (9.0%)	2 (9.0%)	2 (9.0%)	0 (0%)
Age, mean (SD)	21.8 (17)	21.2 (16.5)	21.3 (15.0)	35.1 (26.1)	48.5 (23)	28.5(27.5)
Sex, *n* (%)						
Male	289 (59%)	223 (57%)	58 (68.2%)	5 (71.4%)	4 (57.1%)	0 (0%)
Female	202 (41%)	168 (43%)	27 (31.8%)	2 (28.6%)	3 (42.8%)	2 (100%)

One observation is missing demographic characteristics.

NTS, non-typhoidal *Salmonella*.

### Exploring the time series of *Salmonella*

**Figure 3.**
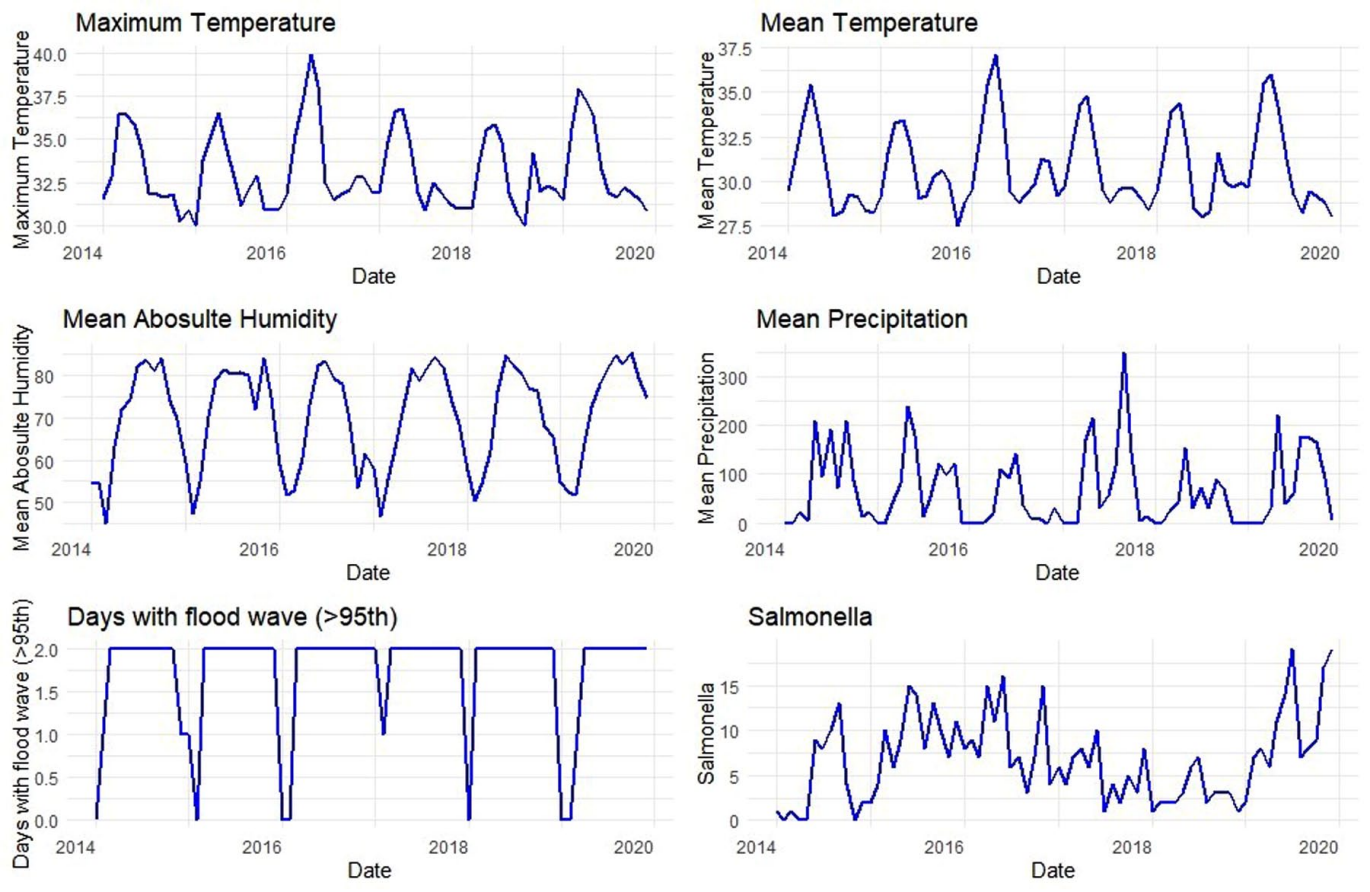
Distribution of monthly meteorological variables and cases of *Salmonella* bacteraemia during the study period, 2014–2019.

**Figure 4.**
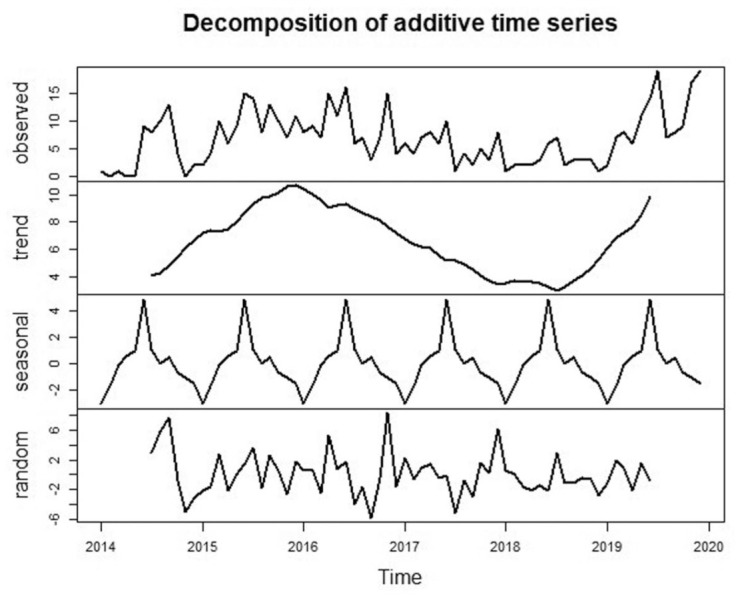
Decomposition of additive time series of *Salmonella.*

The meteorological variables and the notified cases of *Salmonella* bacteraemia were plotted (Figure 3). The time-series decomposition reveals a clear upward trend from 2017 onwards (Figure 4), indicating a rise in *Salmonella* bacteraemia and a strong annual seasonal pattern. The seasonal fluctuations remained consistent throughout the years, while the random component shows some irregular variability, particularly around 2018. The cross-correlation analysis (Figure S1) between *Salmonella* cases and meteorological variables indicates significant relationships. Maximum and mean temperatures and mean absolute humidity show positive correlations with *Salmonella* bacteraemia, particularly at 1–2 lag months. In addition, precipitation shows a short-term effect, with a slight increase in *Salmonella* bacteraemia cases in the current or previous month. Flood events (days with flood wave exceeding the 95th percentile river discharge) exhibit significant positive correlations, with flood conditions contributing to an increase in *Salmonella* bacteraemia in the following 1–3 months.

### Exposure–response–lag relationship

**Figure 5.**
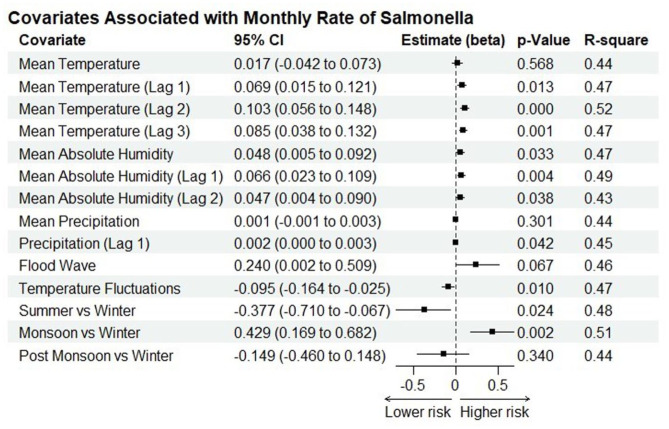
Results from the Quasi-Poisson regression model assessing the impact of various meteorological variables on monthly salmonellosis cases. Each row displays the estimate (beta) and 95% confidence interval (CI) for each covariate, along with the *p*-value and pseudo-*R*^2^.

The preliminary investigation into the associations between meteorological variables and the incidence of *Salmonella* is presented in Figure 5. We observed that an increase in mean temperature with lags of 1–3 months was strongly associated with higher rates of *Salmonella* (Beta = 0.069, *p* = 0.013, *R*^2^ = 0.47), (Beta = 0.103, *p* < .001, *R*^2^ = 0.52) and (Beta = 0.085, *p* = 0.001, *R*^2^ = 0.47), respectively. A similar result was obtained for absolute humidity (AHmean), with current absolute humidity (Beta = 0.048, *p* = 0.033, *R*^2^ = 0.47) and lags 1–2 showing significant positive effects with increased *Salmonella* bacteraemia cases. On the other hand, temperature fluctuations (Beta = −0.095, *p* = 0.010, *R*^2^ = 0.47) showed a significant negative association, while precipitation lagged by 1 month (Beta = 0.0017, *p* = 0.042, *R*^2^ = 0.45) exhibited a small but significant positive association with *Salmonella* rates. There were also seasonal effects on the incidence of *Salmonella*, with the monsoon season (Beta = 0.429, *p* = 0.002, *R*^2^ = 0.51) positively associated with increased cases. By contrast, the summer season was protective (Beta = −0.337, *p* = 0.024, *R*^2^ = 0.48).

**Figure 6.**
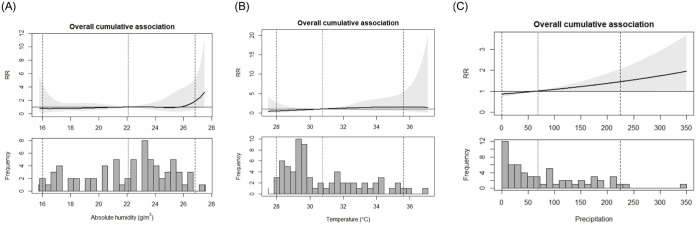
Overall cumulative association between (a) absolute humidity (g/m^3^), (b) temperature (in °C), (c) precipitation and the incidence of *Salmonella* bacteraemia. Top plots: RR with 95% confidence interval. The dark represents the RR, whereas the shaded area represents the 95% confidence interval. Bottom plots: Frequency of *Salmonella* bacteraemia cases. The middle-dotted lines represent the mean values (reference), while the two dashed lines represent the 2.5th and 95th percentile values. The shaded area represents the 95% confidence interval. RR, relative risk.

Figure 6 illustrates the cumulative association between meteorological variables, absolute humidity (g/m³), temperature (°C), precipitation (mm) and the incidence of *Salmonella* bacteraemia. Figure 6(a) shows that the relative risk (RR) of *Salmonella* bacteraemia is lower at absolute humidity levels below 22 g/m³, with a sharp increase observed after 26 g/m³. For example, at the 2.5th percentile of absolute humidity (16 g/m³), the RR of *Salmonella* bacteraemia was 0.76 (95% CI: 0.13, 4.56), while at the 97.5th percentile (26.8 g/m³), the RR was 1.85 (95% CI: 0.65, 5.23). The RR remains relatively constant across lag periods (Figure S2). Similarly, in Figure 6(b), temperature–*Salmonella* associations follow a comparable pattern. At lower temperatures (28°C), the RR was 0.48 (95% CI: 0.08, 2.74), and at higher temperatures (97.5th percentile, 35.6°C), the RR increased to 1.49 (95% CI: 0.41, 5.41). However, it is important to note that none of these results are statistically significant, as indicated by the wide confidence intervals.

The exposure–lag–response relationship (Figure S2) indicates that higher temperatures (above 34°C) are associated with an increased risk, particularly with longer lag periods. The risk of *Salmonella* bacteraemia remains stable at lower precipitation levels but increases significantly beyond 100 mm, with a more pronounced rise after 250 mm (Figure 6(c)). While extreme precipitation is associated with significantly increased risk, such as at the 97.5th percentile (224 mm), where the RR of *Salmonella* bacteraemia was 1.44 (95% CI: 1.01, 2.05), lower precipitation levels are linked to a significantly reduced risk. For instance, at a low precipitation level of 10 mm, the RR was 0.87 (95% CI: 0.76, 0.99). The RR shows a slight upward trend, increasing to about 1.2 at a 1-month lag (Figure S2).

## Discussion

This retrospective study assessed the association between climatic variables and the incidence of *Salmonella* bacteraemia in Mysuru, India. Our findings suggest that extreme climate conditions, particularly absolute humidity and elevated temperatures, may influence the risk of *Salmonella* bacteraemia in this region. Previous studies have documented this positive association primarily in relation to gastroenteritis—four of them measured monthly average temperature and reported a rise in gastroenteritis in England,^20^ Canada,^25^ Macedonia^12^ and China.^25^ Four other studies reported the same association, measuring weekly average temperatures.^21,26–28^ The magnitude of the effect varies depending on the method used to measure meteorological variables, the lag period employed and the region of the world. Only two studies in the United States of America (USA) included positive blood culture cases and reported a positive association with extreme temperature and precipitation events.^13^

There are several explanations for the positive correlation of *Salmonella* bacteraemia and climate variables. *Salmonella* species colonise crop plants when manure is used as an organic fertiliser. Pigs and poultry are typical hosts of *Salmonella*, and their manure is often used as a fertiliser.^29^ Another source of contamination in agriculture is contaminated irrigation water. During extreme climatic events, such as heatwaves and flooding, the scarcity of clean, potable water increases the risk of microbial contamination. A study in Mysore found that although water from the treatment plant met microbiological standards, there was significant isolation of *Escherichia coli* and *Salmonella* when water was stored in containers for an extended period.^30^ A rise in ambient temperature not only increases the risk of exposure to *Salmonella* but also leads to increased virulence and bacteraemia, as we have shown in this study. Genetic analysis of virulence factors in *Salmonella* has shown the differential expression of virulence genes when the bacteria are exposed to heat stress.^31^ These virulence factors enable intracellular entry and survival, colonising the liver and spleen, which are defence organs for bacteremia.^32–34^ Heat stress also compromises gut integrity, facilitating the inflammation and invasiveness of *Salmonella*.^35^

The public health implications of a potential rise in *Salmonella* bacteraemia associated with increasing ambient temperatures due to climate change are enormous in India. As noted in previous studies, *S. typhi* was the most common blood isolate, predominantly affecting males in the 0–10-year age group. Nalidixic acid resistance was 84%, which is similar to the findings reported in the published literature.^7^ A rising trend in ciprofloxacin resistance was also noted. The misuse of antibiotics in animals, the inadequate use of antibiotics in humans and climate change contribute to the increasing resistance to antibiotics in *Salmonella*.

Our study has several strengths. All cases were blood culture-positive, which is the gold standard test for diagnosing *Salmonella* bacteraemia. This is the first study to demonstrate a positive correlation between climate variables and *Salmonella* bacteraemia as a specific outcome. This has been shown through robust statistical modelling using DLNM and generalised additive models, which account for seasonal trends in infections.

We acknowledged the following limitations in this study. Firstly, we did not have data on the residential address of the patients in this study; therefore, we cannot rule out referrals from neighbouring areas. However, the clinician believes that most of the patients attending the hospital reside in Mysore, with very few visiting from outside the district. For those who did, climate variables were unlikely to have significant variations in the surrounding areas. Secondly, water supply quality and distribution can vary seasonally; monsoon periods may increase water contamination, which can confound the association. Thirdly, food safety practices and cold chain systems can break down during extreme weather events, which can contribute to the development of *Salmonella* sepsis. Lastly, this was a single-centre hospital-based study, and the true incidence may be higher, and different effect sizes may be obtained in different climatic conditions. Further studies are needed to explore a causal relationship between climate and *Salmonella* bacteraemia.

## Conclusion

This retrospective, single-centre time-series analysis has shown that maximum monthly ambient temperature, absolute humidity and precipitation are associated with an increase in *Salmonella* bacteraemia. With current trends in climate change in Mysuru, this would result in increased hospitalisation, morbidity and increased use of antibiotics, leading to the risk of resistance. Addressing these needs requires a ‘One-health’ approach to monitoring food and water quality, agricultural practices and disaster management in the event of extreme climatic conditions.

## Supplemental Material

sj-doc-2-tai-10.1177_20499361251389056 – Supplemental material for The impact of meteorological variables on Salmonella bacteraemia in Mysuru District, Karnataka State, India: a retrospective time-series analysis Supplemental material, sj-doc-2-tai-10.1177_20499361251389056 for The impact of meteorological variables on Salmonella bacteraemia in Mysuru District, Karnataka State, India: a retrospective time-series analysis by Naveen Manchal, Mahadevaiah N. Sumana, Megan K. Young, Maria Eugenia Castellanos, Peter Leggat and Oyelola A. Adegboye in Therapeutic Advances in Infectious Disease

sj-docx-1-tai-10.1177_20499361251389056 – Supplemental material for The impact of meteorological variables on Salmonella bacteraemia in Mysuru District, Karnataka State, India: a retrospective time-series analysis Supplemental material, sj-docx-1-tai-10.1177_20499361251389056 for The impact of meteorological variables on Salmonella bacteraemia in Mysuru District, Karnataka State, India: a retrospective time-series analysis by Naveen Manchal, Mahadevaiah N. Sumana, Megan K. Young, Maria Eugenia Castellanos, Peter Leggat and Oyelola A. Adegboye in Therapeutic Advances in Infectious Disease

## Declarations

**Ethics approval and consent to participate:** This project was approved by the JSS Medical College Institutional Ethics Committee with approval number JSSMC/IEC/250325/51 NCT/2024-25 and by James Cook University Human Ethics (H9034) under project HREC/22/QPCH/87676. This is a retrospective study involving patient records. A waiver of consent was approved by the JSS Medical College Institutional Ethics Committee, as there was no direct patient contact and the data were de-identified.

**Consent for publication:** Not applicable.

**Availability of data and materials:** Available on request from the corresponding author.
