# Supplementary material for: The impact of meteorological variables on Salmonella bacteraemia in Mysuru District, Karnataka State, India: a retrospective time-series analysis
Source: Ther Adv Infect Dis. 2025 Oct 27;12:20499361251389056. doi: 10.1177/20499361251389056 (PMC12575929; doi:10.1177/20499361251389056)
Supplement: sj-docx-1-tai-10.1177_20499361251389056 – Supplemental material for The impact of meteorological variables on Salmonella bacteraemia in Mysuru District, Karnataka State, India: a retrospective time-series analysis [file sj-docx-1-tai-10.1177_20499361251389056.docx]

Supplementary material


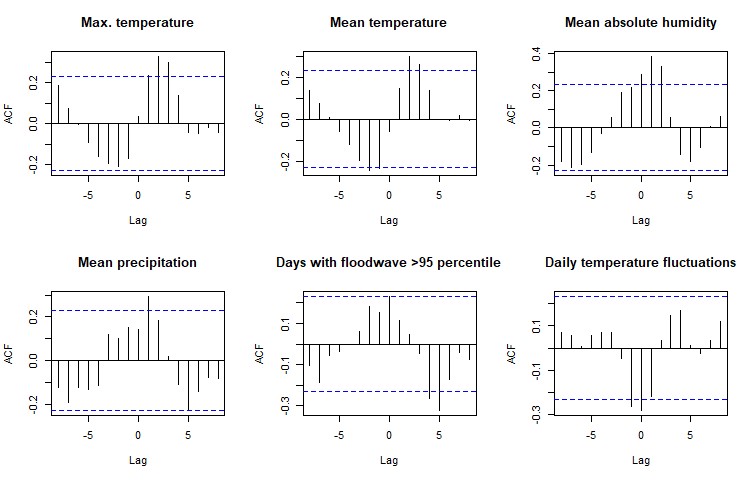


Figure S1. Cross-correlation between *Salmonella* cases and meteorological variables

| 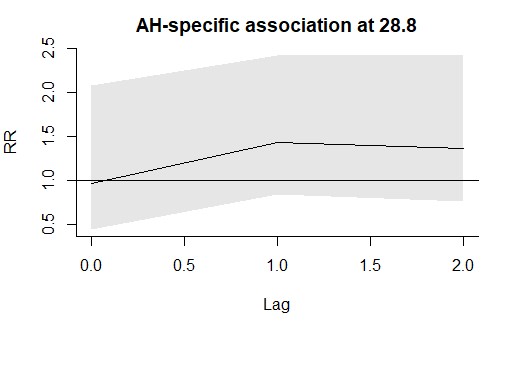 | 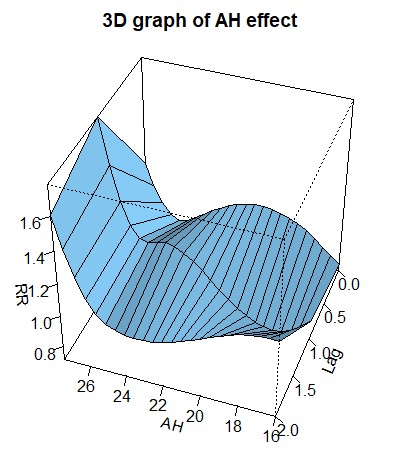 |
| --- | --- |
| 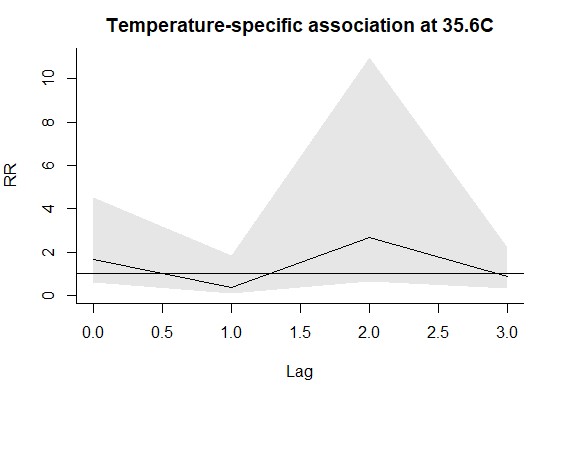 | 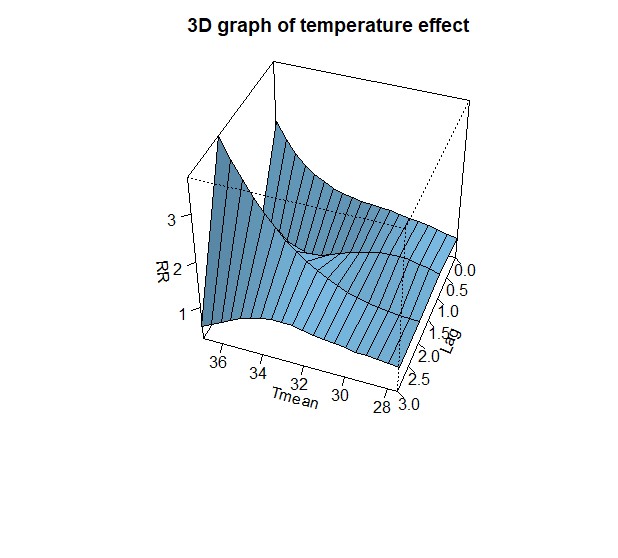 |
| 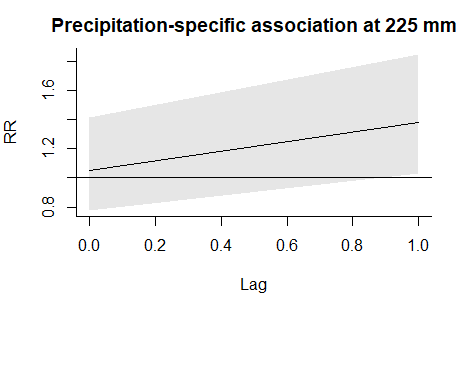 | 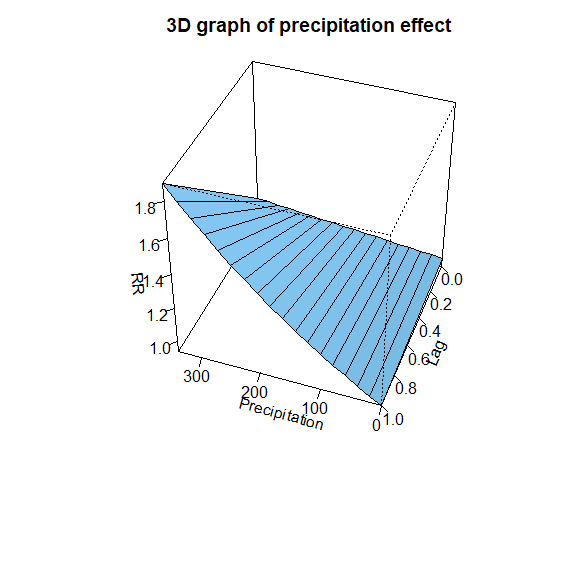 |

Figure S2. Exposure-lag-response relationship for (Top panel) Absolute humidity, (Middle panel) temperature and (Botto panel) precipitation. Right (lag-response relationship), Left (3D association).
